# Gut microbiota: An emerging therapeutic approach of herbal medicine for prevention of colorectal cancer

**DOI:** 10.3389/fcimb.2022.969526

**Published:** 2022-08-16

**Authors:** Hua-Zhong Ying, Wei Xie, Meng-Chuan Wang, Jia-Qi He, Huan-Huan Zhang, Chen-Huan Yu

**Affiliations:** ^1^ Key Laboratory of Experimental Animal and Safety Evaluation, Zhejiang Academy of Medical Sciences (Hangzhou Medical College), Hangzhou, China; ^2^ The First Affiliated Hospital of Zhejiang Chinese Medical University, Hangzhou, China; ^3^ Cancer Hospital of the University of Chinese Academy of Sciences (Zhejiang Cancer Hospital), Hangzhou, China; ^4^ Institute of Cancer and Basic Medicine, Chinese Academy of Sciences, Hangzhou, China

**Keywords:** intestinal homeostasis, natural products, chronic inflammation, probiotic, immunoenhancement, tumor microenvironment

## Abstract

The gut dysbiosis has emerged as a prominent player in the pathogenesis and development of colorectal cancer (CRC), which in turn intensifies dysregulated gut microbiota composition and inflammation. Since most drugs are given orally, this dysbiosis directly and indirectly impinges the absorption and metabolism of drugs in the gastrointestinal tract, and subsequently affects the clinical outcome of patients with CRC. Herbal medicine, including the natural bioactive products, have been used traditionally for centuries and can be considered as novel medicinal sources for anticancer drug discovery. Due to their various structures and pharmacological effects, natural products have been found to improve microbiota composition, repair intestinal barrier and reduce inflammation in human and animal models of CRC. This review summarizes the chemo-preventive effects of extracts and/or compounds derived from natural herbs as the promising antineoplastic agents against CRC, and will provide innovative strategies to counteract dysregulated microbiota and improve the lives of CRC patients.

## 1 Introduction

The incidence of colorectal cancer (CRC) has boosted greatly in the last decades and has become the third leading cause of cancer death, which now accounts for approximately 10% of cancer-related mortality in the world (Bray et al., 2018). The high incidence of CRC has been attributed to the increasingly aging population, unfavorable dietary habits, low physical exercise and excessive obesity (Lund et al., 2011). With the in-depth application of high-throughput sequencing technologies, such as 16S rRNA, metagenomics and metatranscriptomics, in human gut microbiota, emerging evidence has implicated the microbiota in the pathogenesis and prognosis of CRC (Watanabe et al., 2020). Some bacterial species including *Fusobacterium* spp., *Enterococcus* spp., *Escherichia coli*, and *Bacteroides* spp. are most commonly associated with the onset and progression of CRC (Lennard et al., 2016). Changes in microbiota composition (dysbiosis) impair the gut barrier function of epithelial tight junctions and the mucus layer. Consequently, it increases the exposure of the epithelium to bacteria and their toxic metabolites, which may have carcinogenic potential to interfere with cell cycle regulation or directly damage DNA. Bacterial translocation also induces chronic inflammation and triggers a cascade of suppressive immune responses associated with the production of procarcinogens or chemicals such as reactive oxygen species (ROS), bacterial genotoxins (colibactin), and hydrogen sulfide (H_2_S) (Gagnière et al., 2016). In turn, the excessive oxidative stress aggravates colitis and neoplastic processes. Thus, targeting and improving gut microbial dysbiosis could be plausible therapeutic strategies for the prevention and treatment of CRC.

Herbal medicines have been used to prevent and treat diseases for thousands of years which are being developed into decoction and liquid extract for clinical application. When herbal medicines enter the digestive system, they will inevitably come into contact with gut microbes, which could limit excessive inflammatory response and maintain intestinal homeostasis (Chen et al., 2017). Some prodrugs derived from herbal medicines are produced under the metabolism of gut microbes, and subsequently display their antitumor effects to reduce tumor mass and prevent tumorigenesis through several mechanism (Meng et al., 2013; Chen et al., 2016). In addition, bioactive ingredients in herbal medicines may stimulate microorganisms to secrete certain endogenous substances which can enhance barrier stabilization and immune surveillance (Vivarelli et al., 2019). Despite these advances, the underlying molecular mechanism of herbal medicines and their bioactive compounds on microbe-mediated CRC remains extremely deficient. In this review, we highlight the importance of herbal medicine intervention on the intestinal microbiota as an instrument for dysbacteriosis, and consequently, for the prevention of colorectal cancer, suggesting anti-inflammatory, antioxidant and anticarcinogenic properties.

## 2 Gut microbiota and CRC: Potential disease mechanism

**Figure 1:**
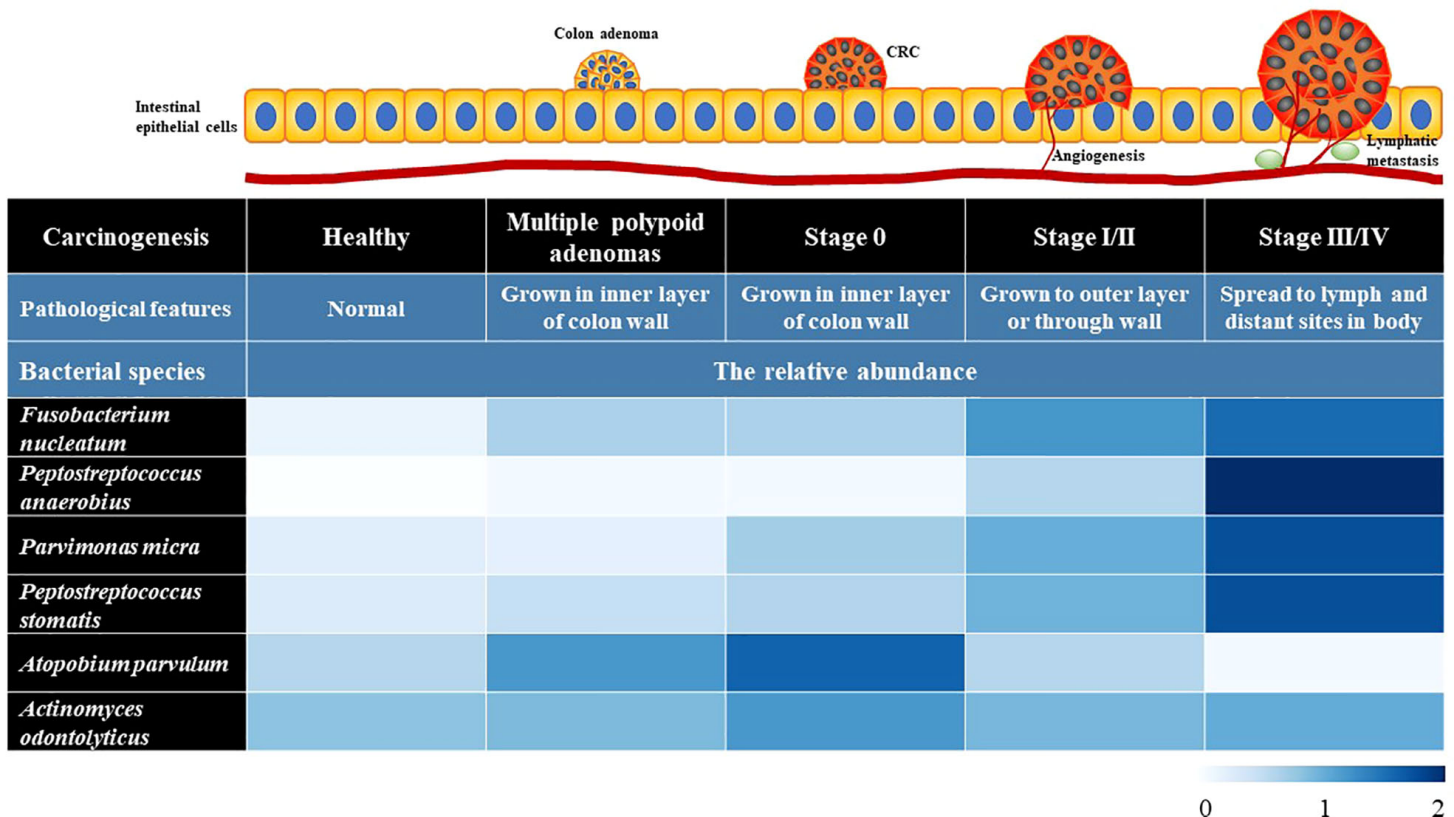
The profiles of gut microbiota in the intestinal tissues of healthy people and the CRC patients at different stages. The data in heatmap were obtained from the reference (Sobhani et al., 2011). The microbiome signature potentially can be used as auxiliary diagnostic biomarkers.

**Figure 2:**
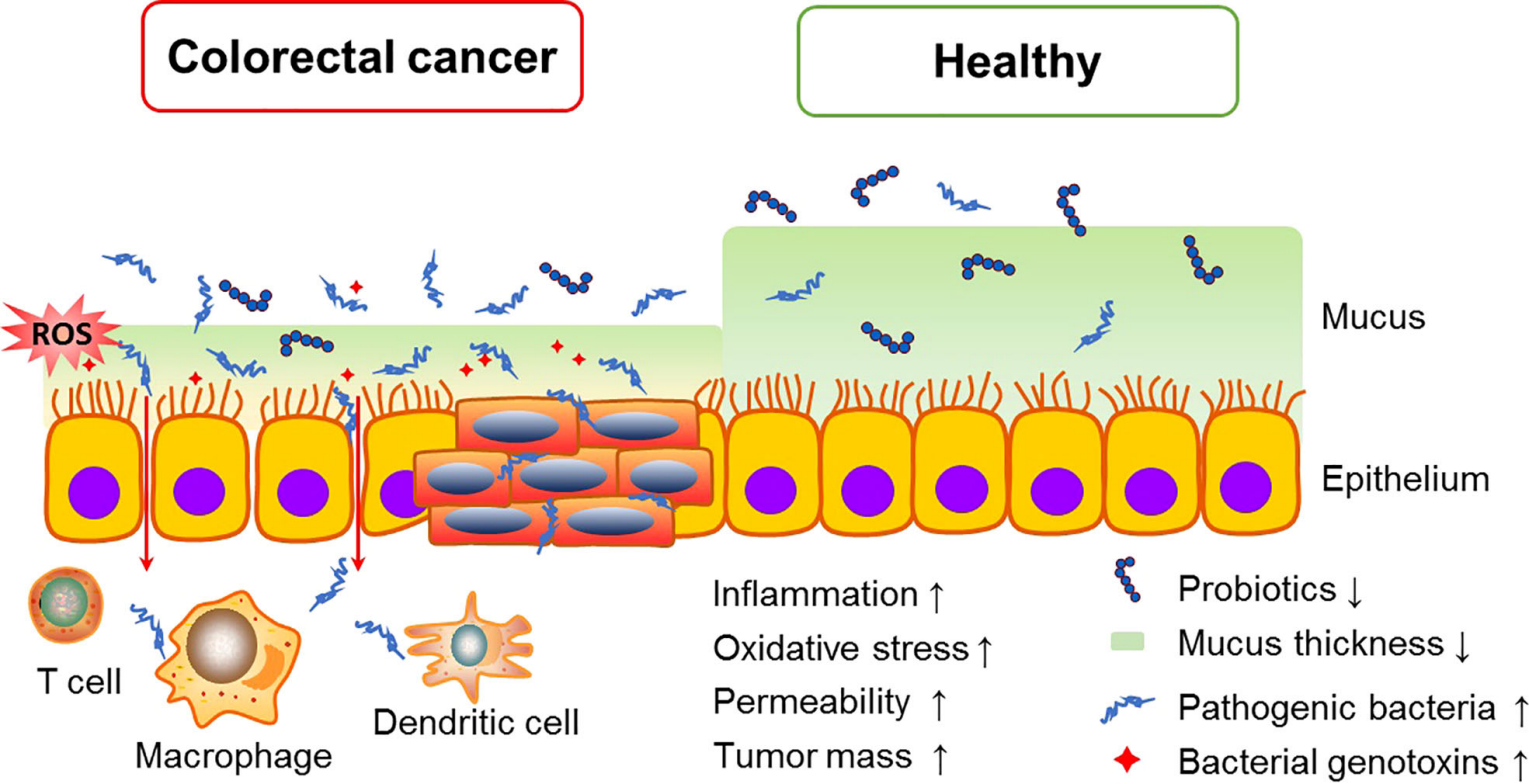
Intestinal dysbiosis accelerate CRC tumorigenesis. Overgrowth of pathogenic bacteria produce toxic metabolites, which can interfere with the cell cycle regulation and directly damage DNA, and also induce chronic inflammation and oxidative stress, consequently promoting CRC growth and spread.

The gut microbiota constitutes a natural defensive barrier to infection. Growing evidence has demonstrated the role of gut microbes in promoting inflammatory responses, creating a suitable microenvironment for the development of skewed interactions between the gut microbiota and cancer initiation (Perillo et al., 2020). Thus, the gut microbiota has been proposed as a novel therapeutic target in light of recent promising data in which it seems to modulate the response to cancer immunotherapy. Moreover, the microbiota involves in numerous protective, structural and metabolic roles in the intestinal epithelium to maintain gut homeostasis. The human intestinal mucosal surface area is more than 200 m^2^. There are about 10^3^ different microorganisms, which are 10-fold more than the total number of human cells (Sekirov et al., 2010). More than 3×10^7^ genes in the gut microbiota are considered as the second genome of humans, and approximately 10% of the metabolite cycles occur in the human intestinal micro-ecological environment (Gill et al., 2006; Wu et al., 2013). CRC is frequently associated with dramatic alterations in the microbial composition of the tumor and adjacent mucosa (**Figure 1**). Clinical trials proved that the abundance of *Fusobacterium nucleatum* started at stage 0 and increased as the CRC progressed, while *Atopobium parvulum* and *Actinomyces odontolyticus* were significantly increased in patients with multiple polypoid adenomas and/or stage 0 but no longer increased in more advanced stages, *Peptostreptococcus anaerobius*, *Peptostreptococcus stomatis*, and *Parvimonas micra* increased at stage I~IV (Sobhani et al., 2011). In addition, the number of beneficial bacteria such as *Bifidobacterium*, *Helicobacter oxysporum*, and *Haemophilus* was reduced from the polyps to CRC stage 0 (Mizutani et al., 2020). Although the causal relationship between gut dysbiosis and CRC remains unclear, gut dysbiosis exacerbates the development of colorectal cancer mainly *via* intestinal inflammation, immunotolerance, and oxidative stress (**Figure 2**).

### 2.1 Inhibition of intestinal inflammation

Inflammations caused by gut microbes are the main mechanisms to induce tumorigenesis (Konstantinov et al., 2013). The disorder of gut microbiota induces the hyperpermeability of the intestinal wall, and then helps the pathogenic bacteria and its endotoxin to break out into the bloodstream, resulting in chronic inflammatory response and immuno-suppression (Avril and DePaolo, 2021). Chronic inflammation reshapes the tumor microenvironment, and promote tumorigenesis and even metastasis *via* activating numerous exogenous and endogenous signaling pathways (Yu and Fang, 2015). Pathogen recognition receptors (PPRs) are a series of innate immune receptors mainly including toll-like receptors (TLRs) and Nod-like receptors (NLRs). Once the intestinal epithelial barrier is breached, PPRs rapidly sense nucleic acids and antigen components from bacteria and subsequently induce the secretion of type I interferon and antimicrobial peptides to defense against intestinal pathogens. However, inappropriately vigorous innate immune responses can also activate cell survival signaling mainly *via* NF-κB, STAT3 and MAPK pathways, which elevate the transcription of pro-inflammatory cytokines. Then secondary enteric inflammatory challenges prolong systemic inflammation and expedite proliferation and metastasis of tumor cells (Fukata and Abreu, 2009). Indeed, the patients with inflammatory bowel disease (IBD) presented an increased risk of developing CRC (Feagins et al., 2009; Rogler, 2014). In those patients, the number of probiotics such as *Lactobacillus* and *Bifidobacteria* is reduced, while *Parvimonas micra*, Phascolarctobacterium, *Streptococcus bovis* and *S. gallolyticus* are increased (Uronis et al., 2009; Richard et al., 2018). Oral administration of antibiotics, probiotic preparation or antioxidants significantly decrease the number of mucosal nodules and suppress colon tumorigenesis in the azoxymethane (AOM) and dextran sulfate sodium (DSS)-induced CRC mouse model (Hattori et al., 2019; Luo et al., 2019), suggesting that altering mucosa-associated bacterial microbiota and chronic inflammation in the IBD patients may be beneficial for CRC prevention.

### 2.2 Regulation of bacterial enzymes and metabolites

Gut microbiota is also involved in the production of various enzymes and metabolites. During gastrointestinal tumorigenesis, the physiological capacities of several bacteria are changed, resulting in the odd levels of bacterial enzymes and their metabolites. Bacterial enzymes including β-glucuronidase, nitroso-reductase, nitrate reductase, β-glucosidase, azo-reductase and 7α-dehydrooxygenase from gut microbiota disorders can induce the alteration of intestinal metabolites (such as secondary bile acids and H_2_S), thereby producing various carcinogens and promoting the occurrence of colorectal cancer (Azcárate-Peril et al., 2011). Primary bile acids excreted into the gut are converted into secondary bile acids which can increase reactive oxygen species through microbial derived-metabolism, such as hydrolase, leading to DNA damage and genomic instability, and finally induce the growth of tumors (Saracut et al., 2015). Clostridium converts primary bile acid into deoxycholic acid, increasing free radicals and ROS to induce chronic inflammation and colorectal cancer. H_2_S is a metabolite produced by sulfate-reducing bacteria in the gut tract which can cause DNA damage, free radical release, colonic mucosal inflammation and hyperplasia, suppress cytochrome oxidase and DNA methylation, and ultimately contribute to tumor initiation (Wang et al., 2021).

### 2.3 Reduction of oxidative stress

ROS is also blamed as being a driving force behind CRC initiation. *Enterococcus faecalis* releases extracellular superoxide, and after transformed by hydrogen peroxide, these free radicals as powerful mutagens can cause DNA breakage, and local genomic instability in CRC patients with colorectal cancer (Kabwe et al., 2021; Rivas-Domínguez et al., 2021). Similarly, *Helicobacter pylori* promotes the development of gastrointestinal inflammation and carcinogenesis *via* elevating ROS and reactive nitrogen species (RNS) to upregulate oncogenic pathways such as HIF-1α, NF-κB and PI3K/AKT (Liu et al., 2019; Lu et al., 2020). Therefore, targeting those pathogenic bacteria or counteract their deleterious effects (reducing ROS generation) have been considered as potential strategies for preventing CRC.

## 3 Herbal medicine, gut microbiota and colorectal cancer

**Figure 3:**
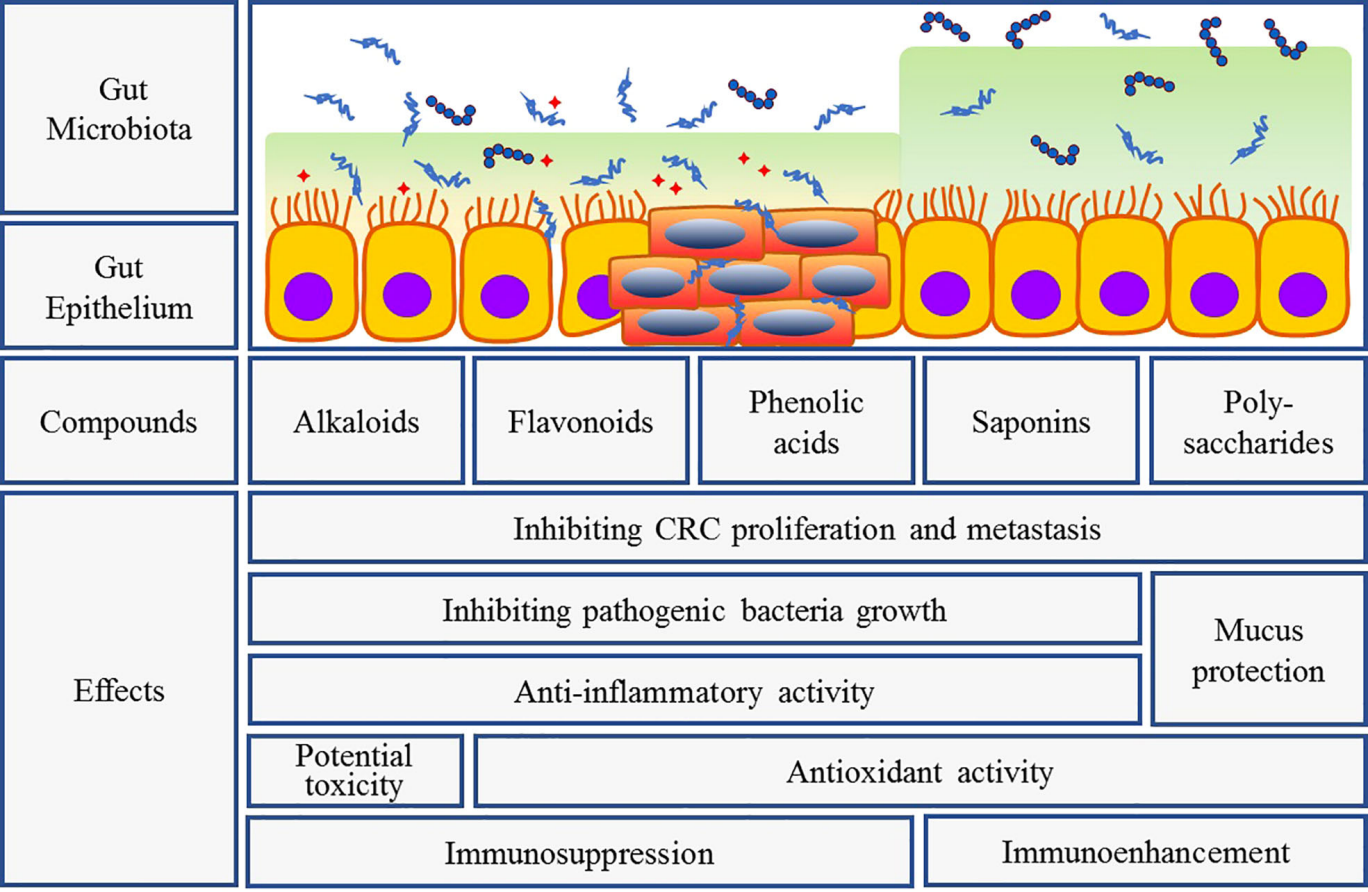
Intervention of herbal bioactive components on gut bacteria and CRC. Interaction between herbal medicine and gut microbiota can fight tumor growth and prevent tumorigenesis through several mechanisms: (1) inhibiting pathogenic bacteria overgrowth and promoting probiotics growth; (2) anti-inflammatory and antioxidant activities as well as intestinal mucosal protection and immune regulation; (3) direct anti-tumor activity. But different components exert their respective features.

Herbal medicines including herbal formulas, extracts, and compounds have been studied for many years in the treatment of gut-related diseases *via* regulating gut micro-ecosystem. It can apparently alter the composition and metabolism of gut microbiota, and dramatically affect the number and function of intestinal epithelial cells to achieve the rebalancing of gut microecology (**Figure 3**) (Li et al., 2021).

### 3.1 Regulation of gut microbiota and metabolites by herbal medicine

Herbal medicines are rich in chemical constituents which contains not only bioactive ingredients such as glycosides, flavonoids, alkaloids, quinones and steroids, but also nutrients such as protein and vitamins, leading to the variety of their pharmacodynamic effects. The mechanism of herbal medicine in improving gut microbiota imbalance includes two aspects: inhibiting pathogenic bacterial overgrowth and promoting probiotics growth. At present, CRC-associated probiotics are mainly divided into three categories: *Lactobacillus*, *Bifidobacteriums*, and Gram-positive cocci. On the one hand, probiotics indirectly inhibit the growth and invasion of pathogenic bacteria through strengthening the barrier function of intestinal epithelial cells and producing beneficial metabolites (Kaur et al., 2021). On the other hand, probiotics have physiologically positive effects on the host (animals or human) through regulating the immune function of the host mucosa and system, or improving the balance of gut nutrition and microbiota composition. What’s more, probiotics also play a role in inhibiting allergies, controlling serum cholesterol level, and regulating immune function such as metabolic transformation and metabolic detoxification for preventing the gastrointestinal carcinogenesis (Paveljšek et al., 2021).

Many herbal medicines, such as *Ganoderma Lucidum*, American Ginseng, Red Ginseng, *Gynostemma Pentaphyllum*, and Curcumin, significantly inhibit the growth of pathogenic bacteria such as *Clostridium*, *Escherichia*, *Staphylicus*, *Verrucomicrobia* but increase the number of probiotic *Bifidobacteria* and *lactobacilli*, resulting in the increased diversity of the gut microbiota in DSS-induced CRC mouse model (Guo et al., 2015; McFadden et al., 2015; Yu et al., 2015; Chen et al., 2016; Wang et al., 2016; Gong et al., 2019; Lv et al., 2019 ;Jiang et al., 2020; Shen et al., 2020; Sui et al., 2020; Sun et al., 2020 ;Zhang et al., 2020; Hao et al., 2021; Wang et al., 2021; Zhang et al., 2021; Zhu et al., 2021). These anti-CRC profiles of herbal medicine on gut microbiota are summarized as shown in **Table 1**.

**Table 1:** Regulation of herbal medicine on gut microbiota changes in CRC-related animal models.

Herbal name	Animal model	Gut Microbiota change	Anti-CRC mechanism	Reference(s)
Red Ginseng (Radix Et Rhizoma Ginseng)	Trinitro–Benzene–Sulfonic acid induced ulcerative colitis Wistar rats	Bifidobacteriu↑ Lactobacillu↑ E.coli↓	Promotes probiotic growth; inhibits pathogenic bacteria growth	(Zhu et al., 2021)
American Ginseng (Radix Panacis Quinquefolii)	AOM/DSS-induced colitis and colon carcinogenesis A/J mice	Firmicutes↑ Verrucomicrobia ↓	Inhibits inflammatory cytokines; inhibits pathogenic bacteria growth	(Wang et al., 2021)
Gynostemma Leaf (Gynostemmatis Pentaphylli Folium)	C57BL/6J-Apc^Min/+^ mice	Sulfate-reducing bacteria↓	Inhibits pathogenic bacteria growth; modulates inflammatory intestinal microenvironment.	(Zhang et al., 2021)
Curcumin	AOM-induced colitis and colon cancer IL10^−/−^ mice	Lactobacillus↑ Coriobacterales↓	Promotes probiotic growth; inhibits pathogenic bacteria growth	(Hao et al., 2021)
Berberine	DMH-induced colon cancer mice	Fusobacterium nucleatum↓ Tenericutes↓ Verrucomicrobia↓	Inhibits pathogenic bacteria growth; increases the secretion of IL-21/22/31 and CD40L; up-regulates the expression of p-STAT3, p-STAT5 and p-ERK1/2.	(Zhang et al., 2020)
Yi-Yi-Fu-Zi-Bai-Jiang-San (YYFZBJS)	C57BL/6J-Apc^Min/+^ mice	Bacteroides fragilis Lachnospiraceae	Reduces Intestinal lymphatic, and mesenteric lymph nodes, accumulated CD4^+^ CD25^+^ Foxp3^+^ Treg cells, along with reduction of the phosphorylation of β-catenin.	(Shen et al., 2020)
Wu Mei Wan (WMW)	AOM/DSS-induced CAC mouse	Bacteroidetes↓ Bacteroidales_s24-7_group↓ Firmicutes↑ Lachnospiraceae↑	Regulates the balance between “tumor-promoting bacteria” and “tumor-suppressing bacteria” and inactivated the NF-κB/IL-6/STAT3 pathway.	(Sun et al., 2020)
Gegen Qinlian decoction (GOD)	Patients with CRC	Megamonas↓ Veillonella↓ Bacteroides↑ Akkermansia↑ Prevotella↑	Promotes probiotic growth; inhibits pathogenic bacteria growth.	(La et al., 2017)
Neohesperidin (NHP)	Apc^Min/+^ mouse	Bacteroidetes↓ Firmicutes↑ Proteobacteria↑	Promotes probiotic growth; inhibits pathogenic bacteria growth.	(Huang et al., 2017)
Evodiamine (EVO)	AOM/DSS-induced CAC mouse	Enterococcus faecalis↓ Escherichia col↓ Bifidobacterium↑ Campylobacter↑ Lactobacillus↑	Promotes probiotic growth; inhibits pathogenic bacteria growth; inhibits the IL6/STAT3/P65 signaling pathway.	(Li et al., 2017; Zhu et al., 2019)
Pai-Nong-San (PNS)	AOM/DSS-induced CAC mouse	Firmicutes↑ Bacteroidetes↓ Proteobacteria↑ Lactobacillus↑	Regulates the expression of CD4+ and CD8+ T cells; inhibits the production of HIF-α, IL-6, and TNF-α; promotes the expression of IL-4 and IFN-γ in colon tissues; improves gut microbiota; inhibits the Wnt signaling pathway.	(He et al., 2016)
Xiaoyaosan (XYS)	CRC xenografts mice	Bacteroides Lactobacillus Desulfovibrio Rikenellaceae.	Promotes probiotic growth; inhibits pathogenic bacteria growth.	(Kawabata et al., 2012; Song et al., 2018)
Huangqin-tea (HQT)	Pseudo-germ-free rat model	Lachnoclostridium↑ Alistipes↑ Roseburia↑ Lactococcus↑ Bacteroides↓ Parasutterella ↓ Clostridiales↓.	Decreases IL-1β, IL-6, IL-10, and TNF-α expression; elevates IFN-γ production; Promotes probiotic growth; inhibits pathogenic bacteria growth.	(Noratto et al., 2011)
Quxie capsule	Patients with metastatic colorectal cancer	g_Bifidobacterium ↓ Collinsella ↓ Ruminiclostridium_9 ↓	Enhances CD4+ cells among mCRC patients; increase the abundance of gut anticancer bacteria Actinobacteria and butyrate-producing bacteria Lachnospiraceae.	(Angel-Morales et al., 2012)

AOM, azoxymethane; DSS, dextran sulfate sodium; DMH, 1,2-dimethylhydrazine; SCFAs, short-chain fatty acids; TCM, traditional Chinese medicine; APC, adenomatosis polyposis coli; ↑, upregulate. ↓downregulate.

### 3.2 Anti-inflammatory and intestinal mucosal immunity of herbal bioactive ingredients

It has been known that chronic intestinal inflammation is closely related to gut microenvironment, and gut inflammation mostly leads to colorectal cancer (Konstantinov et al., 2013; Avril and DePaolo, 2021). Under physiological conditions, both gut bacteria and viruses can’t transmit through the mucosa. However, when inflammatory and neoplastic intestinal disorders exist, the permeability of intestinal barrier will increase, causing higher translocation of bacteria and viruses into the bloodstream. The active ingredients of herbal medicines can alleviate the stimulating effect of gut microbiota on tumors by improving the tumor microenvironment, such as inflammation and immunosuppression, thus inhibiting the development of tumors and even the metastasis and recurrence after operation.

*Coptidis Rhizoma* (also called as Huanglian), is derived from the rhizome of Coptis chinensis Franch., Coptis deltoidea C.F. Cheng et Hisao or Coptis teeta Wall. Berberine is a main activealkaloid isolated and identified from this herb. Evidences support the folkloric medicinal properties of *Coptidis Rhizoma*, in particular berberine, as a promising anticancer candidate in CRC *via* inducing AMPK activation and autophagic cell death (Huang et al., 2017; La et al., 2017). Berberine mitigates intestinal inflammation and oxidant stress through blocking the IL-6/STAT3, Nrf2 and PPAR pathways on colitis-associated tumorigenesis in mice (Li et al., 2017; Zhu et al., 2019). Moreover, it can not only significantly increase the abundance of Brucella, Bacteroides, Clostridium butyricum and Helicobacter in gut tract, but also protects intestinal epithelial cells against CRC-induced intestinal barrier dysfunction (Zhu et al., 2019).

Genistein is the predominant isoflavone found in Leguminous plants (such as *Sophora japonica L*. and *Glycine max*) and acts as the strong tyrosine kinase inhibitor, topoisomerase inhibitor and PPARγ agonist. It exerts phytoestrogenic, antioxidant and anti-inflammatory effects on gut microenvironment *via* regulating COX-2-related signaling pathway in CRC mice (He et al., 2016; Song et al., 2018). Crocin, a natural carotenoid from saffron (*Croci stigma*) and gardenia (*Gardeniae fructus*), can inhibit the expression of pro-inflammatory cytokines and inducible inflammatory enzymes in AOM/DSS-induced colorectal inflammation model, and significantly reduce inflammation and mucosal ulcer (Kawabata et al., 2012). The flavanol-rich foods as well as red wine polyphenolics inhibited ROS generation and NF-κB activation in colon cells by inducing miR-126 and miR-146a (Noratto et al., 2011; Angel-Morales et al., 2012).

FCT, a synbiotic combination of probiotic Lactobacillus gasseri 505 (LG) and Cudrania tricuspidata leaf extract (CT), reduced the risk of colitis-associated colon cancer *via* regulating inflammation, carcinogenesis, and gut microbiota composition. Compared with CT and LG, FCT significantly down-regulated pro-inflammatory mediators (TNF-α, IFN-γ, IL-1β, IL-6, iNOS and COX-2), and up-regulated anti-inflammatory cytokines (IL-4 and IL-10). In addition, FTC enhanced gut barrier function *via* up-regulating mucus layer markers (MUC-2 and TFF3) and tight junction (occludin and ZO-1), decreasing *Staphylococcus* and increasing *Lactobacillus*, *Bifidobacterium*, and *Akkermansia*, resulting in the increased production of short-chain fatty acids (SCFAs) (Oh et al., 2020).

Most notably, clinical responses to immune checkpoint inhibitors are closely associated with the abnormal gut microbiome composition, especially the relative abundance of *Akkermansia muciniphila* (Routy et al., 2018). Although several herbal medicines had been demonstrated to prevent the CRC growth, reduce side effects of chemotherapy and enhance the efficacy of PD-1 inhibitors *via* modulating the gut microbiota composition and CD4+ T cell proportion in tumor beds (Lv et al., 2019; Zhang et al., 2021; Huang et al., 2022; Messaoudene et al., 2022), the key orchestrators responsible for the primary resistance to PD-1 blockers remain unclear.

### 3.3 Improving bioavailability of herbal medicine by gut microbiota

Gut microbiota modifies the chemical composition of herbal medicine through their own enzymatic system. Intestinal cells also influence the metabolism and absorption of herbal medicine through transporter proteins and metabolic enzymes. After underwent by gut microbiota biotransformation (including hydrolysis, oxidation and reduction reaction), the chemical composition, pharmacological activity and toxicity of the herbal medicines will be changed, and it will form new active metabolites. Therefore, the biotransformation induced by gut microbiota has a central impact on exerting the efficiency of herbal medicine (Shen et al., 2013).

#### 3.3.1 Glycosides

Most glycosides, including saponins, flavonoids and anthraquinones, will be hydrolyzed by gut microbiota to remove glycosyl groups and form aglycones, which reduces polarity, increases lipo-solubility and facilitates absorption into the blood. Both licorice and ginseng contain saponins. Glycyrrhetinic acid could be detected in normal rats after oral administration of glycyrrhizin, but not in sterile rats, indicating that gut microbiota could be converted into glycyrrhetinic acid and then absorbed by organisms (Takeda et al., 1996). Gut microbiota can promote the absorption and metabolic transformation of ginsenoside Rb1 and ginsenoside Rd, which can promote the biosynthesis of RNA and protein, regulate body metabolism and enhance immune function (Kim et al., 2014). Compound K, the main metabolite of ginseng saponin, induces apoptosis of colorectal cancer cells by inhibiting histone deacetylase activity (Kang et al., 2013). Most flavonoids, such as baicalin and isoquercitrin, are α-glucosylated by gut microbiota, but those enzymatical modification enhances their intestinal absorption and pharmacological action (Wang et al., 2015; Lee et al., 2017; Terao, 2017). More notably, degraded by *Clostridium orbiscindens*, diet flavonoids were cracked and converted into desaminotyrosine, which can up-regulate the signal pathway of type I interferon and enhance the host antiviral immune response (Steed et al., 2017).

#### 3.3.2 Alkaloids

Alkaloids are a class of nitrogen-containing organic compounds, which have strong pharmacological activities in the central nervous system, cardiovascular system, immune function, anti-bacterial, anti-inflammatory and anti-cancer. As like as flavonoids, many alkaloids, such as berberine, aconitine and scopolamine, are usually characterized by small molecules, or by ether bonds and coordination bond which are prone to hydrolysis and dehydration under the action of gut microbiota. Under the action of gut bacteria, aconitine, a poisonous alkaloid mainly obtained from *Aconitum carmichaeli Debxwhich* shows chondroprotective activity can produce new monoester, diester and lipid alkaloids through deacetylation, demethylation, dehydroxylation and esterification, which greatly reduces the toxicity of aconitine and alleviates intestinal irritation (Tong et al., 2014). α-Chaconine, a potato glycoside alkaloid, induces apoptosis of HT-29 colon cancer cells by activating caspase-3 and inhibiting ERK 1/2 phosphorylation, and inhibits *Enterobacter aerogenes*, *Escherichia coli* and *Staphylococcus aureus* (Yang et al., 2006).

#### 3.3.3 Phenylpropanoids

Phenylpropanoids generally have lactone structure, including phenylpropionic acid, coumarin and lignans. After the catalysis of gut microbiota, lactone structure can be broken or demethylated. The metabolic transformation of silymarin in Eubacterium limosum produced demethylsilybin A, demethylsilybin B, demethylisosilybin A and demethylisosilybin B which had stronger inhibitory effects on Alzheimer’s amyloid protein-β42 (Zhang et al., 2014). Proteasome degraded flaxseed lignans in human intestinal tract bacteria. Hydrolysis and deglycosylation removed two sugars to form isopine resin diol diester (SECO). Pine resin diol diester was produced by digestive Streptococcus *Petostreptococcus productus*, *Eubacterium limosum*, *Clodium methoxybenzo-vorans* and *Eggelentala tarda* under the action of digestive *peptococcus*, *Eubacterium limosum*, *Clodium methoxybenzo-vorans* and *Eggetala tarda.* They were demethylated and dehydroxylated to form enterediol and enterolactone (Eeckhaut et al., 2008; Woting et al., 2010; Mabrok et al., 2012). A study of the metabolic mechanism of lignans in flaxseed, demonstrated that Ruminal Prevotella was the main microbial group for lignans metabolism (Schogor et al., 2014).

## 4 Conclusion and perspectives

Herbal medicines contain a variety of bioactive compounds and have unique advantages on maintenance of intestinal homeostasis and regulation of host immune. It precisely regulates the microbiota composition to indirectly prevent the CRC occurrence and development. On the other hand, the active ingredients in herbal medicine can directly inhibit the growth of colon cancer cells. Gut microorganisms produce many metabolic enzymes during their growth and reproduction, such as hydrolase, lyase, transferase and redox enzymes, which improve the bioavailability of the effective components of herbal medicine by biotransformation. Many active ingredients of Chinese herbal medicines can be transformed by gut microorganisms to produce metabolites with strong pharmacological effects, which can be easily absorbed by the body and exert anticancer activity. Thus, herbal medicine has the promise of preventing or delaying CRC progression *via* maintenance of intestinal homeostasis.

However, it should be pointed out that, in terms of current research, there is no evidence that herbal medicine can cure tumors only by improving gut microbiota, or more by exerting direct effects on cancer cells. Secondly, it is difficult to determine the sequence of the effects of herbal medicine and gut microbiota, just like the problem of “eggs and chickens” which one appears first. Although some specific bacteria that cause a precancerous phenotype *in vivo* have been identified, whether gut dysbiosis is the culprit behind CRC rather than a result of inflammation is still in dispute. Thus, deep signal regulation pathways and key targets of gut microbiota as a potent herbal medicine intervention in colorectal cancer need to be further explored.

## Author contributions

H-ZY, WX, and M-CW searched the articles and drafted the manuscript. J-QH and H-HZ checked the contents. H-ZY and C-HY revised the manuscript. C-HY was responsible for the project administration and funding acquisition. All authors contributed to the article and approved the submitted version.

## Funding

This work is supported by National Natural Science Foundation of China (No. 82104526) and Zhejiang province of medical science (No. 2022KY917).

## Conflict of interest

The authors declare that the research was conducted in the absence of any commercial or financial relationships that could be construed as a potential conflict of interest.

## Publisher’s note

All claims expressed in this article are solely those of the authors and do not necessarily represent those of their affiliated organizations, or those of the publisher, the editors and the reviewers. Any product that may be evaluated in this article, or claim that may be made by its manufacturer, is not guaranteed or endorsed by the publisher.

## Abbreviations

AMPK, adenosine 5’-monophosphate-activated protein kinase; APC, adenomatosis polyposis coli; AOM, azoxymethane; CAC, colitis associated cancer; COX2, cyclo-oxygenase-2; CRC, colorectal cancer; DSS, dextran sodium sulfate; FOXO3, forkhead box O3; IL-17, interleukin 17; MAPK, mitogen-activated protein kinase; NF-κB, nuclear factor kappa-B; PI3K, phosphoinositide 3-kinase; TGF-β1, transforming growth factor-β1; ROS, reactive oxygen species; SCFAs, short chain fatty acids; STAT3, signal transducer and activator of transcription 3.

## References

[B1] Angel-Morales G. Noratto G. Mertens-Talcott S. (2012). Red wine polyphenolics reduce the expression of inflammation markers in human colon-derived CCD-18Co myofibroblast cells: Potential role of microRNA-126. Food Funct. 3 (7), 745–752. doi: 10.1039/c2fo10271d 22572890

[B2] Avril M. DePaolo R. W. (2021). "Driver-passenger" bacteria and their metabolites in the pathogenesis of colorectal cancer. Gut Microbes 13 (1), 1941710. doi: 10.1080/19490976.2021.1941710 34225577 PMC8265790

[B3] Azcárate-Peril M. A. Sikes M. Bruno-Bárcena J. M. (2011). The intestinal microbiota, gastrointestinal environment and colorectal cancer: A putative role for probiotics in prevention of colorectal cancer. Am. J. Physiol. Gastrointest Liver Physiol. 301 (3), G401–G424. doi: 10.1152/ajpgi.00110.2011 21700901 PMC3774253

[B4] Bray F. Ferlay J. Soerjomataram I. Siegel R. L. Torre L. A. Jemal A. (2018). Global cancer statistics 2018: GLOBOCAN estimates of incidence and mortality worldwide for 36 cancers in 185 countries. CA Cancer J. Clin. 68 (6), 394–424. doi: 10.3322/caac.21492 30207593

[B5] Chen L. Brar M. S. Leung F. C. Hsiao W. L. (2016). Triterpenoid herbal saponins enhance beneficial bacteria, decrease sulfate-reducing bacteria, modulate inflammatory intestinal microenvironment and exert cancer preventive effects in ApcMin/+ mice. Oncotarget 7 (21), 31226–31242. doi: 10.18632/oncotarget.8886 27121311 PMC5058752

[B6] Chen F. Jiang J. Tian D. D. Wen Q. Li Y. H. Zhang J. Q.. (2017). Targeting obesity for the prevention of chronic cardiovascular disease through gut microbiota-herb interactions: An opportunity for traditional herbs. Curr. Pharm. Des. 23 (8), 1142–1152. doi: 10.2174/1381612822666161014115724 27758701

[B7] Chen F. Wen Q. Jiang J. Li H. L. Tan Y. F. Li Y. H.. (2016). Could the gut microbiota reconcile the oral bioavailability conundrum of traditional herbs. J. Ethnopharmacol. 179, 253–264. doi: 10.1016/j.jep.2015.12.031 26723469

[B8] Eeckhaut E. Struijs K. Possemiers S. Vincken J. P. Keukeleire D. D. Verstraete W. (2008). Metabolism of the lignan macromolecule into enterolignans in the gastrointestinal lumen as determined in the simulator of the human intestinal microbial ecosystem. J. Agric. Food Chem. 56 (12), 4806–4812. doi: 10.1021/jf800101s 18494490

[B9] Feagins L. A. Souza R. F. Spechler S. J. (2009). Carcinogenesis in IBD: potential targets for the prevention of colorectal cancer. Nat. Rev. Gastroenterol. Hepatol. 6 (5), 297–305. doi: 10.1038/nrgastro.2009.44 19404270

[B10] Fukata M. Abreu M. T. (2009). Pathogen recognition receptors, cancer and inflammation in the gut. Curr. Opin. Pharmacol. 9 (6), 680–687. doi: 10.1016/j.coph.2009.09.006 19828376 PMC2826797

[B11] Gagnière J. Raisch J. Veziant J. Barnich N. Bonnet R. Buc E.. (2016). Gut microbiota imbalance and colorectal cancer. World J. Gastroenterol. 22 (2), 501–518. doi: 10.3748/wjg.v22.i2.501 26811603 PMC4716055

[B12] Gill S. R. Pop M. Deboy R. T. Eckburg P. B. Turnbaugh P. J. Samuel B. S.. (2006). Metagenomic analysis of the human distal gut microbiome. Science 312 (5778), 1355–1359. doi: 10.1126/science.1124234 16741115 PMC3027896

[B13] Gong Y. Dong R. Gao X. Li J. Jiang L. Zheng J.. (2019). Neohesperidin prevents colorectal tumorigenesis by altering the gut microbiota. Pharmacol. Res. 148, 104460. doi: 10.1016/j.phrs.2019.104460 31560944

[B14] Guo M. Ding S. Zhao C. Gu X. He X. Huang K.. (2015). Red ginseng and semen coicis can improve the structure of gut microbiota and relieve the symptoms of ulcerative colitis. J. Ethnopharmacol. 162, 7–13. doi: 10.1016/j.jep.2014.12.029 25554637

[B15] Hao W. Wu J. Yuan N. Gong L. Huang J. Ma Q.. (2021). Xiaoyaosan improves antibiotic-induced depressive-like and anxiety-like behavior in mice through modulating the gut microbiota and regulating the NLRP3 inflammasome in the colon. Front. Pharmacol. 12. doi: 10.3389/fphar.2021.619103 PMC8087337 33935710

[B16] Hattori N. Niwa T. Ishida T. Kobayashi K. Imai T. Mori A.. (2019). Antibiotics suppress colon tumorigenesis through inhibition of aberrant DNA methylation in an azoxymethane and dextran sulfate sodium colitis model. Cancer Sci. 110 (1), 147–156. doi: 10.1111/cas.13880 30443963 PMC6317928

[B17] He X. Bai Y. Zhao Z. Wang X. Fang J. Huang L.. (2016). Local and traditional uses, phytochemistry, and pharmacology of sophora japonica l.: A review. J. Ethnopharmacol. 187, 160–182. doi: 10.1016/j.jep.2016.04.014 27085938

[B18] Huang J. Liu D. Wang Y. Liu L. Li J. Yuan J.. (2022). Ginseng polysaccharides alter the gut microbiota and kynurenine/tryptophan ratio, potentiating the antitumour effect of antiprogrammed cell death 1/programmed cell death ligand 1 (anti-PD-1/PD-L1) immunotherapy. Gut 71 (4), 734–745. doi: 10.1136/gutjnl-2020-321031 34006584 PMC8921579

[B19] Huang T. Xiao Y. Yi L. Li L. Wang M. Tian C.. (2017). Coptisine from rhizoma coptidis suppresses HCT-116 cells-related tumor growth *in vitro* and *in vivo* . Sci. Rep. 7, 38524. doi: 10.1038/srep38524 28165459 PMC5292956

[B20] Jiang F. Liu M. Wang H. Shi G. Chen B. Chen T.. (2020). Wu Mei wan attenuates CAC by regulating gut microbiota and the NF-kB/IL6-STAT3 signaling pathway. BioMed. Pharmacother. 125, 109982. doi: 10.1016/j.biopha.2020.109982 32119646

[B21] Kabwe M. Meehan-Andrews T. Ku H. Petrovski S. Batinovic S. Chan H. T.. (2021). Lytic bacteriophage EFA1 modulates HCT116 colon cancer cell growth and upregulates ROS production in an enterococcus faecalis Co-culture system. Front. Microbiol. 12. doi: 10.3389/fmicb.2021.650849 PMC8044584 33868210

[B22] Kang K. A. Piao M. J. Kim K. C. Zheng J. Yao C. W. Cha J. W.. (2013). Compound K, a metabolite of ginseng saponin, inhibits colorectal cancer cell growth and induces apoptosis through inhibition of histone deacetylase activity. Int. J. Oncol. 43 (6), 1907–1914. doi: 10.3892/ijo.2013.2129 24100442

[B23] Kaur H. Gupta T. Kapila S. Kapila R. (2021). Protective effects of potential probiotic lactobacillus rhamnosus (MTCC-5897) fermented whey on reinforcement of intestinal epithelial barrier function in a colitis-induced murine model. Food Funct. 12 (13), 6102–6116. doi: 10.1039/d0fo02641g 34047732

[B24] Kawabata K. Tung N. H. Shoyama Y. Sugie S. Mori T. Tanaka T. (2012). Dietary crocin inhibits colitis and colitis-associated colorectal carcinogenesis in Male ICR mice. Evid. Based. Complement Alternat. Med. 2012, 820415. doi: 10.1155/2012/820415 23326291 PMC3543809

[B25] Kim K. A. Yoo H. H. Gu W. Yu D. H. Jin M. J. Choi H. L.. (2014). Effect of a soluble prebiotic fiber, NUTRIOSE, on the absorption of ginsenoside Rd in rats orally administered ginseng. J. Ginseng Res. 38 (3), 203–207. doi: 10.1016/j.jgr.2014.03.003 25378995 PMC4213839

[B26] Konstantinov S. R. Kuipers E. J. Peppelenbosch M. P. (2013). Functional genomic analyses of the gut microbiota for CRC screening. Nat. Rev. Gastroenterol. Hepatol. 10 (12), 741–745. doi: 10.1038/nrgastro.2013.178 24042452

[B27] La X. Zhang L. Li Z. Yang P. Wang Y. (2017). Berberine-induced autophagic cell death by elevating GRP78 levels in cancer cells. Oncotarget 8 (13), 20909–20924. doi: 10.18632/oncotarget.14959 28157699 PMC5400555

[B28] Lee Y. S. Woo J. B. Ryu S. I. Moon S. K. Han N. S. Lee S. B. (2017). Glucosylation of flavonol and flavanones by bacillus cyclodextrin glucosyltransferase to enhance their solubility and stability. Food Chem. 229, 75–83. doi: 10.1016/j.foodchem.2017.02.057 28372240

[B29] Lennard K. S. Goosen R. W. Blackburn J. M. (2016). Bacterially-associated transcriptional remodelling in a distinct genomic subtype of colorectal cancer provides a plausible molecular basis for disease development. PloS One 11 (11), e0166282. doi: 10.1371/journal.pone.0166282 27846243 PMC5112903

[B30] Liu I. L. Tsai C. H. Hsu C. H. Hu J. M. Chen Y. C. Tian Y. F.. (2019). Helicobacter pylori infection and the risk of colorectal cancer: A nationwide population-based cohort study. QJM 112 (10), 787–792. doi: 10.1093/qjmed/hcz157 31250012

[B31] Li X. Wu D. Niu J. Sun Y. Wang Q. Yang B.. (2021). Intestinal flora: A pivotal role in investigation of traditional Chinese medicine. Am. J. Chin. Med. 49 (2), 237–268. doi: 10.1142/S0192415X21500130 33622213

[B32] Li D. Zhang Y. Liu K. Zhao Y. Xu B. Xu L.. (2017). Berberine inhibits colitis-associated tumorigenesis *via* suppressing inflammatory responses and the consequent EGFR signaling-involved tumor cell growth. Lab. Invest. 97 (11), 1343–1353. doi: 10.1038/labinvest.2017.71 28759012

[B33] Lund E. K. Belshaw N. J. Elliott G. O. Johnson I. T. (2011). Recent advances in understanding the role of diet and obesity in the development of colorectal cancer. Proc. Nutr. Soc. 70 (2), 194–204. doi: 10.1017/S0029665111000073 21385524

[B34] Luo S. Wen R. Wang Q. Zhao Z. Nong F. Fu Y.. (2019). Rhubarb peony decoction ameliorates ulcerative colitis in mice by regulating gut microbiota to restoring Th17/Treg balance. J. Ethnopharmacol. 231, 39–49. doi: 10.1016/j.jep.2018.08.033 30170079

[B35] Lu Y. Rong J. Lai Y. Tao L. Yuan X. Shu X. (2020). The degree of helicobacter pylori infection affects the state of macrophage polarization through crosstalk between ROS and HIF-1α. Oxid. Med. Cell Longev. 2020, 5281795. doi: 10.1155/2020/5281795 33376580 PMC7746446

[B36] Lv J. Jia Y. Li J. Kuai W. Li Y. Guo F.. (2019). Gegen qinlian decoction enhances the effect of PD-1 blockade in colorectal cancer with microsatellite stability by remodelling the gut microbiota and the tumour microenvironment. Cell Death Dis. 10 (6), 415. doi: 10.1038/s41419-019-1638-6 31138779 PMC6538740

[B37] Mabrok H. B. Klopfleisch R. Ghanem K. Z. Clavel T. Blaut M. Loh G. (2012). Lignan transformation by gut bacteria lowers tumor burden in a gnotobiotic rat model of breast cancer. Carcinogenesis 33 (1), 203–208. doi: 10.1093/carcin/bgr256 22080573

[B38] McFadden R. M. Larmonier C. B. Shehab K. W. Midura-Kiela M. Ramalingam R. Harrison C. A.. (2015). The role of curcumin in modulating colonic microbiota during colitis and colon cancer prevention. Inflamm Bowel Dis. 21 (11), 2483–2494. doi: 10.1097/MIB.0000000000000522 26218141 PMC4615313

[B39] Meng Q. X. Roubin R. H. Hanrahan J. R. (2013). Ethnopharmacological and bioactivity guided investigation of five TCM anticancer herbs. J. Ethnopharmacol. 148 (1), 229–238. doi: 10.1016/j.jep.2013.04.014 23623820

[B40] Messaoudene M. Pidgeon R. Richard C. Ponce M. Diop K. Benlaifaoui M.. (2022). A natural polyphenol exerts antitumor activity and circumvents anti-PD-1 resistance through effects on the gut microbiota. Cancer Discov 12 (4), 1070–1087. doi: 10.1158/2159-8290.CD-21-0808 35031549 PMC9394387

[B41] Mizutani S. Yamada T. Yachida S. (2020). Significance of the gut microbiome in multistep colorectal carcinogenesis. Cancer Sci. 111 (3), 766–773. doi: 10.1111/cas.14298 31910311 PMC7060472

[B42] Noratto G. D. Kim Y. Talcott S. T. Mertens-Talcott S. U. (2011). Flavonol-rich fractions of yaupon holly leaves (Ilex vomitoria, aquifoliaceae) induce microRNA-146a and have anti-inflammatory and chemopreventive effects in intestinal myofibroblast CCD-18Co cells. Fitoterapia 82 (4), 557–569. doi: 10.1016/j.fitote.2011.01.013 21262328

[B43] Oh N. S. Lee J. Y. Kim Y. T. Kim S. H. Lee J. H. (2020). Cancer-protective effect of a synbiotic combination between lactobacillus gasseri 505 and a cudrania tricuspidata leaf extract on colitis-associated colorectal cancer. Gut Microbes 12 (1), 1785803. doi: 10.1080/19490976.2020.1785803 32663105 PMC7524312

[B44] Paveljšek D. Ivičak-Kocjan K. Treven P. Benčina M. Jerala R. Rogelj I. (2021). Distinctive probiotic features share common TLR2-dependent signalling in intestinal epithelial cells. Cell Microbiol. 23 (1), e13264. doi: 10.1111/cmi.13264 32945079 PMC7757178

[B45] Perillo F. Amoroso C. Strati F. Giuffrè M. R. Díaz-Basabe A. Lattanzi G.. (2020). Gut microbiota manipulation as a tool for colorectal cancer management: Recent advances in its use for therapeutic purposes. Int. J. Sci. 21 (15), 5389. doi: 10.3390/ijms21155389 PMC7432108 32751239

[B46] Richard M. L. Liguori G. Lamas B. Brandi G. da Costa G. Hoffmann T. W.. (2018). Mucosa-associated microbiota dysbiosis in colitis associated cancer. Gut Microbes 9 (2), 131–142. doi: 10.1080/19490976.2017.1379637 28914591 PMC5989788

[B47] Rivas-Domínguez A. Pastor N. Martínez-López L. Colón-Pérez J. Bermúdez B. Orta M. L. (2021). The role of DNA damage response in dysbiosis-induced colorectal cancer. Cells 10 (8), 1934. doi: 10.3390/cells10081934 34440703 PMC8391204

[B48] Rogler G. (2014). Chronic ulcerative colitis and colorectal cancer. Cancer Lett. 345 (2), 235–241. doi: 10.1016/j.canlet.2013.07.032 23941831

[B49] Routy B. Le Chatelier E. Derosa L. Duong C. P. M. Alou M. T. Daillère R.. (2018). Gut microbiome influences efficacy of PD-1-based immunotherapy against epithelial tumors. Science 359 (6371), 91–97. doi: 10.1126/science.aan3706 29097494

[B50] Saracut C. Molnar C. Russu C. Todoran N. Vlase L. Turdean S.. (2015). Secondary bile acids effects in colon pathology. Exp. Mice Study Acta Cir. Bras. 30 (9), 624–631. doi: 10.1590/S0102-865020150090000007 26465107

[B51] Schogor A. L. Huws S. A. Santos G. T. Scollan N. D. Hauck B. D. Winters A. L.. (2014). Ruminal prevotella spp. may play an important role in the conversion of plant lignans into human health beneficial antioxidants. PloS One 9 (4), e87949. doi: 10.1371/journal.pone.0087949 24709940 PMC3977842

[B52] Sekirov I. Russell S. L. Antunes L. C. Finlay B. B. (2010). Gut microbiota in health and disease. Physiol. Rev. 90 (3), 859–904. doi: 10.1152/physrev.00045.2009 20664075

[B53] Shen H. Leung W. I. Ruan J. Q. Li S. L. Lei J. P. Wang Y. T.. (2013). Biotransformation of ginsenoside Rb1 *via* the gypenoside pathway by human gut bacteria. Chin. Med. 8 (1), 22. doi: 10.1186/1749-8546-8-22 24267405 PMC4175505

[B54] Shen J. Li P. Liu S. Liu Q. Li Y. Zhang Z.. (2020). The chemopreventive effects of huangqin-tea against AOM-induced preneoplastic colonic aberrant crypt foci in rats and omics analysis. Food Funct. 11 (11), 9634–9650. doi: 10.1039/d0fo01731k 33048099

[B55] Sobhani I. Tap J. Roudot-Thoraval F. Roperch J. P. Letulle S. Langella P.. (2011). Microbial dysbiosis in colorectal cancer (CRC) patients. PloS One 6 (1), e16393. doi: 10.1371/journal.pone.0016393 21297998 PMC3029306

[B56] Song S. Cheng D. Wei S. Wang X. Niu Y. Qi W.. (2018). Preventive effect of genistein on AOM/DSS-induced colonic neoplasm by modulating the PI3K/AKT/FOXO3 signaling pathway in mice fed a high-fat diet. J. Funct. Foods 46, 237–242. doi: 10.1016/j.jff.2018.05.006

[B57] Steed A. L. Christophi G. P. Kaiko G. E. Sun L. Goodwin V. M. Jain U.. (2017). The microbial metabolite desaminotyrosine protects from influenza through type I interferon. Science 357 (6350), 498–502. doi: 10.1126/science.aam5336 28774928 PMC5753406

[B58] Sui H. Zhang L. Gu K. Chai N. Ji Q. Zhou L.. (2020). YYFZBJS ameliorates colorectal cancer progression in ApcMin/+ mice by remodeling gut microbiota and inhibiting regulatory T-cell generation. Cell Commun. Signal. 18 (1), 113. doi: 10.1186/s12964-020-00596-9 32677955 PMC7367414

[B59] Sun L. Yan Y. Chen D. Yang Y. (2020). Quxie capsule modulating gut microbiome and its association with T cell regulation in patients with metastatic colorectal cancer: Result from a randomized controlled clinical trial. Integr. Cancer Ther. 19, 1534735420969820. doi: 10.1177/1534735420969820 33243018 PMC7876934

[B60] Takeda S. Ishthara K. Wakui Y. Amagaya S. Maruno M. Akao T.. (1996). Bioavailability study of glycyrrhetic acid after oral administration of glycyrrhizin in rats; relevance to the intestinal bacterial hydrolysis. J. Pharm. Pharmacol. 48 (9), 902–905. doi: 10.1111/j.2042-7158.1996.tb05998.x 8910850

[B61] Terao J. (2017). Factors modulating bioavailability of quercetin-related flavonoids and the consequences of their vascular function. Biochem. Pharmacol. 139, 15–23. doi: 10.1016/j.bcp.2017.03.021 28377278

[B62] Tong P. Xu S. Cao G. Jin W. Guo Y. Cheng Y.. (2014). Chondroprotective activity of a detoxicated traditional Chinese medicine (Fuzi) of aconitum carmichaeli debx against severe-stage osteoarthritis model induced by mono-iodoacetate. J. Ethnopharmacol. 151 (1), 740–744. doi: 10.1016/j.jep.2013.11.048 24315981

[B63] Uronis J. M. Mühlbauer M. Herfarth H. H. Rubinas T. C. Jones G. S. Jobin C. (2009). Modulation of the intestinal microbiota alters colitis-associated colorectal cancer susceptibility. PloS One 4 (6), e6026. doi: 10.1371/journal.pone.0006026 19551144 PMC2696084

[B64] Vivarelli S. Salemi R. Candido S. Falzone L. Santagati M. Stefani S.. (2019). Gut microbiota and cancer: From pathogenesis to therapy. Cancers (Basel) 11 (1), 38. doi: 10.3390/cancers11010038 PMC6356461 30609850

[B65] Wang Y. Nguyen L. H. Mehta R. S. Song M. Huttenhower C. Chan A. T. (2021). Association between the sulfur microbial diet and risk of colorectal cancer. JAMA Netw. Open 4 (11), e2134308. doi: 10.1001/jamanetworkopen.2021.34308 34767023 PMC8590167

[B66] Wang C. Z. Yu C. Wen X. D. Chen L. Zhang C. F. Calway T.. (2016). American Ginseng attenuates colitis-associated colon carcinogenesis in mice: Impact on gut microbiota and metabolomics. Cancer Prev. Res. (Phila) 9 (10), 803–811. doi: 10.1158/1940-6207.CAPR-15-0372 27443884 PMC5052115

[B67] Wang C. Z. Zhang C. F. Chen L. Anderson S. Lu F. Yuan C. S. (2015). Colon cancer chemopreventive effects of baicalein, an active enteric microbiome metabolite from baicalin. Int. J. Oncol. 47 (5), 1749–1758. doi: 10.3892/ijo.2015.3173 26398706 PMC4599184

[B68] Wang M. Zhou B. Cong W. Zhang M. Li Z. Li Y.. (2021). Amelioration of AOM/DSS-induced murine colitis-associated cancer by evodiamine intervention is primarily associated with gut microbiota-Metabolism-Inflammatory signaling axis. Front. Pharmacol. 12. doi: 10.3389/fphar.2021.797605 PMC8740177 35002731

[B69] Watanabe D. Murakami H. Ohno H. Tanisawa K. Konishi K. Tsunematsu Y.. (2020). Association between dietary intake and the prevalence of tumourigenic bacteria in the gut microbiota of middle-aged Japanese adults. Sci. Rep. 10 (1), 15221.doi: 10.1038/s41598-020-72245-7 32939005 PMC7495490

[B70] Woting A. Clavel T. Loh G. Blaut M. (2010). Bacterial transformation of dietary lignans in gnotobiotic rats. FEMS Microbiol. Ecol. 72 (3), 507–514. doi: 10.1111/j.1574-6941.2010.00863.x 20370826

[B71] Wu N. Yang X. Zhang R. Li J. Xiao X. Hu Y.. (2013). Dysbiosis signature of fecal microbiota in colorectal cancer patients. Microb. Eco. 66 (2), 462–470. doi: 10.1007/s00248-013-0245-9 23733170

[B72] Yang S. A. Paek S. H. Kozukue N. Lee K. R. Kim J. A. (2006). Alpha-chaconine, a potato glycoalkaloid, induces apoptosis of HT-29 human colon cancer cells through caspase-3 activation and inhibition of ERK 1/2 phosphorylation. Food Chem. Toxicol. 44 (6), 839–846. doi: 10.1016/j.fct.2005.11.007 16387404

[B73] Yu Y. N. Fang J. Y. (2015). Gut microbiota and colorectal cancer. Gastrointest. Tumors 2 (1), 26–32. doi: 10.1159/000380892 26674881 PMC4668798

[B74] Yu Y. N. Yu T. C. Zhao H. J. Sun T. T. Chen H. M. Chen H. Y.. (2015). Berberine may rescue fusobacterium nucleatum-induced colorectal tumorigenesis by modulating the tumor microenvironment. Oncotarget 6 (31), 32013–32026. doi: 10.18632/oncotarget.5166 26397137 PMC4741656

[B75] Zhang S. L. Mao Y. Q. Zhang Z. Y. Li Z. M. Kong C. Y. Chen H. L.. (2021). Pectin supplement significantly enhanced the anti-PD-1 efficacy in tumor-bearing mice humanized with gut microbiota from patients with colorectal cancer. Theranostics 11 (9), 4155–4170. doi: 10.7150/thno.54476 33754054 PMC7977465

[B76] Zhang Z. Shao S. Zhang Y. Jia R. Hu X. Liu H.. (2020). Xiaoyaosan slows cancer progression and ameliorates gut dysbiosis in mice with chronic restraint stress and colorectal cancer xenografts. BioMed. Pharmacother. 132, 110916. doi: 10.1016/j.biopha.2020.110916 33113425

[B77] Zhang Y. Yang D. H. Zhang Y. T. Chen X. M. Li L. L. Cai S. Q. (2014). Biotransformation on the flavonolignan constituents of silybi fructus by an intestinal bacterial strain eubacterium limosum ZL-II. Fitoterapia 92, 61–71. doi: 10.1016/j.fitote.2013.10.001 24125915

[B78] Zhang M. M. Yin D. K. Rui X. L. Shao F. P. Li J. C. Xu L.. (2021). Protective effect of pai-Nong-San against AOM/DSS-induced CAC in mice through inhibiting the wnt signaling pathway. Chin. J. Nat. Med. 19 (12), 912–920. doi: 10.1016/S1875-5364(22)60143-2 34961589

[B79] Zhu L. Gu P. Shen H. (2019). Protective effects of berberine hydrochloride on DSS-induced ulcerative colitis in rats. Int. Immunopharmacol. 68, 242–251. doi: 10.1016/j.intimp.2018.12.036 30743078

[B80] Zhu L. Q. Zhang L. Zhang J. Chang G. L. Liu G. Yu D. D.. (2021). Evodiamine inhibits high-fat diet-induced colitis-associated cancer in mice through regulating the gut microbiota. J. Integr. Med. 19 (1), 56–65. doi: 10.1016/j.joim.2020.11.001 33277208

